# Light Quality Plays a Crucial Role in Regulating Germination, Photosynthetic Efficiency, Plant Development, Reactive Oxygen Species Production, Antioxidant Enzyme Activity, and Nutrient Acquisition in Alfalfa

**DOI:** 10.3390/ijms26010360

**Published:** 2025-01-03

**Authors:** Md Atikur Rahman, Sang-Hoon Lee, Hyung Soo Park, Chang-Woo Min, Jae Hoon Woo, Bo Ram Choi, Md. Mezanur Rahman, Ki-Won Lee

**Affiliations:** 1 Grassland and Forages Division, National Institute of Animal Science, Rural Development Administration, Cheonan 31000, Republic of Korea; atik.rahmanbt@gmail.com (M.A.R.); sanghoon@korea.kr (S.-H.L.); anpark69@korea.kr (H.S.P.); mcw1004@korea.kr (C.-W.M.); delpire@korea.kr (J.H.W.); qaz8715@korea.kr (B.R.C.); 2 ABEx Bio-Research Center, Dhaka 1230, Bangladesh; 3 Department of Plant and Soil Science, Institute of Genomics for Crop Abiotic Stress Tolerance, Texas Tech University, Lubbock, TX 79409, USA; mdmerahm@ttu.edu

**Keywords:** red-blue light, ROS, antioxidant defense, agronomic traits, *Medicago sativa*

## Abstract

Light is a vital regulator of photosynthesis, energy production, plant growth, and morphogenesis. Although these key physiological processes are well understood, the effects of light quality on the pigment content, oxidative stress, reactive oxygen species (ROS) production, antioxidant defense systems, and biomass yield of plants remain largely unexplored. In this study, we applied different light-emitting diode (LED) treatments, including white light, red light, blue light, and a red+blue (1:1) light combination, to evaluate the traits mentioned above in alfalfa (*Medicago sativa* L.). Fluorescence staining showed that red light significantly triggered the oxidative stress indicators compared to blue and white light, while the combined red and blue light treatment significantly reduced the ROS (O_2_^•−^, H_2_O_2_) intensity in alfalfa seedlings. Interestingly, the combined light treatment significantly boosted the seed germination rate (%), maximum photochemical quantum yield of PSII (Fv/Fm), leaf greenness (SPAD score), photosynthetic pigment levels (chlorophyll a, chlorophyll b, and carotenoids), and plant biomass yield in alfalfa seedlings. The red and/or combined (red+blue) light treatments significantly regulated antioxidant enzymes (SOD, CAT, APX, and GR) and the expression of genes related to the ascorbate–glutathione (AsA-GSH) pathway, including monodehydroascorbate reductase (*MsMDHAR*), dehydroascorbate reductase (*MsDHAR*), ascorbate peroxidase (*MsAPX*), and glutathione reductase (*MsGR*). These results indicate that light quality is crucial for regulating the morphological, physiological, and molecular traits linked to alfalfa improvement. These findings suggest a new approach to enhancing the adaptation, as well as the morphological and agronomic yield, of alfalfa and forage legumes through light-quality-mediated improvement.

## 1. Introduction

Light is a vital source for plant growth and development, driving key physiological processes such as photosynthesis, energy production, nutrient absorption, and the effective use of nutrients within plants [1]. Light quality influences the photosynthetic system, and enhancement of the quality light ratio (red:blue; R:B) significantly boosts the maximal chlorophyll a (Chl a) fluorescence of plants during both their light and dark adaptation [2]. An optimal combination of light (R:B) produces high biomass and metabolite production in plants [2]. In natural environments, plants adapt by using their light-sensing traits to distinguish between sunlight and dark, gray shade on the soil layer and green shade beneath the canopy [3]. Notably, light sensing enables plants to perceive environmental cues regarding the day’s duration and time [4]. However, the high intensity of artificial light results in challenges for plants, and the physiological and molecular mechanisms that govern quality-light-mediated growth and nutrient acquisition in forage plants are largely unknown. Therefore, a fine-tuned strategy to improving the nutrient quality in alfalfa is highly desirable, and is a key target of this research. In this context, LEDs are frequently used to enhance plant growth and function. LEDs that emit narrow-band light influence various aspects of plant biology, including plants’ seed germination, growth, photosynthesis, photosynthetic apparatus (PSA), and nutrition acquisition [2,3,5]. However, it is necessary to explore the research gaps on how light intensity and quality affect ROS signaling, antioxidant enzyme activity, associated gene expression, and mineral nutrition acquisitions in horticulture and other plant species.

Blue light (BL), red light (RL), and far-red light (FRL) induce reactive oxygen species (ROS) generation in response to light intensity in several plant species. For instance, Borbély et al. found that BL and yellow light induce high levels of hydrogen peroxide (H_2_O_2_), superoxide radicals (O_2_^•−^), and malondialdehyde (MDA) in *Camptotheca acuminata* compared to white light (WL) and RL [6]. In contrast, Chinese cabbage exhibited lower levels of H_2_O_2_ in response to BL than in response to WL exposure [7]. Additionally, Chai et al. found that RL significantly induces H_2_O_2_ and programmed cell death (PCD), which are mediated by the redox-sensitive protein LSD1 and HY5 in *Arabidopsis* [8]. These studies suggest that ROS levels are differentially regulated in plants according to their received light intensity and quality.

Improving plant growth, metabolite production, and ROS regulation through light quality is closely associated with the activation of key antioxidant enzymes in plants [9]. Zha et al. found that red:blue (1:3) light increased the amounts of the enzymes MDHAR, DHAR, and GR in lettuce following 12 days of continuous light exposure (200 μmol·m^−2^·s^−1^), while the transcript levels of GMP, GME, GGP, GPP, GLDH, APX, MDHAR, DHAR, and GR were elevated after 3 days of light exposure [10]. The same study noted that the elevation of AsA pool-related enzymes is linked to the blue light intensity received by plants. However, the ratio of blue, red, or even FR is crucial for adjusting the ROS and antioxidant levels in plants. For instance, a low R:FR ratio increased the ROS and MDA levels even under control conditions in tomato plants, while a combined light treatment reduced ROS formation under salt stress [11]. In contrast, a low level of R:FR light reduced salt-induced cellular damage by increasing the activity and gene expression of the SOD, CAT, APX, and POD enzymes [12,13]. However, questions remain about how individual and/or combined exposure to red or blue regulates ROS signals, antioxidant enzyme activity, candidate gene expression, and nutrient acquisition in other crops like legumes or forage species.

Several studies have documented that nutrient acquisition and utilization are altered by the light quality and intensity of light received by plants. For instance, blue light induces the accumulation of N, P, and K in garlic leaves [14]. Red light combined with blue light increases the accumulation of Ca, Zn, Cu, Fe, and Se, but not the accumulation of Mn and P in *Gynostemma pentaphyllum* [15]. However, blue light alone enhances the levels of Cu, Zn, Fe, and Mg in lettuce leaves [16]. Combined red and blue light (1:4) improved the acquisition of Cu, Fe, S, Zn, B, P, Mg, Ca, Mg, and Mo in broccoli [17]. Despite these advancements in horticulture plants, further research is needed to understand how the quality of light can influence the germination, photosynthesis, ROS signals, gene expression, and mineral acquisition in legumes and forage plants.

Alfalfa (*Medicago sativa* L.), often referred to as the “queen of forage”, is widely cultivated as a forage legume. Nutrition deficiencies can adversely affect its growth, development, plant biomass, and digestibility, thus impacting livestock productivity [18]. Imposing quality light-mediated improvements to enhance the forage yield, nutrient quality, plant fitness, and adaptation of alfalfa would be an alternative approach to forage plant development. This study, therefore, aimed to explore the role of light quality on the photosynthetic efficiency, yield traits, oxidative stress markers, antioxidant enzyme activity, candidate gene expression, and nutrient acquisition/uptake in alfalfa seedlings.

## 2. Results

### 2.1. Improved Germination, Morphological and Physiological Traits

**Figure 1:**
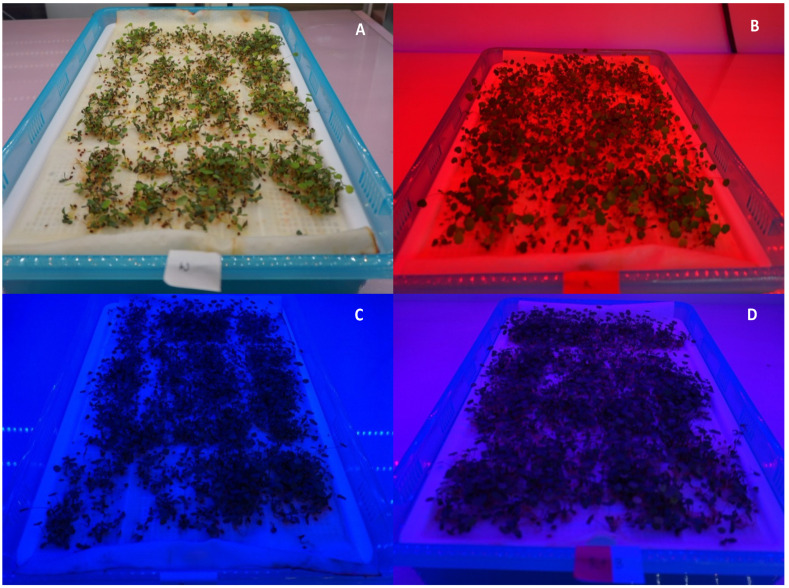
Phenotypic variation in alfalfa seedlings under different light qualities. Alfalfa seedlings are grown under white (**A**), red (**B**), blue (**C**), and red combined with blue light (red+blue) (**D**). Photographs are taken after two weeks of seedling growth.

**Figure 2:**
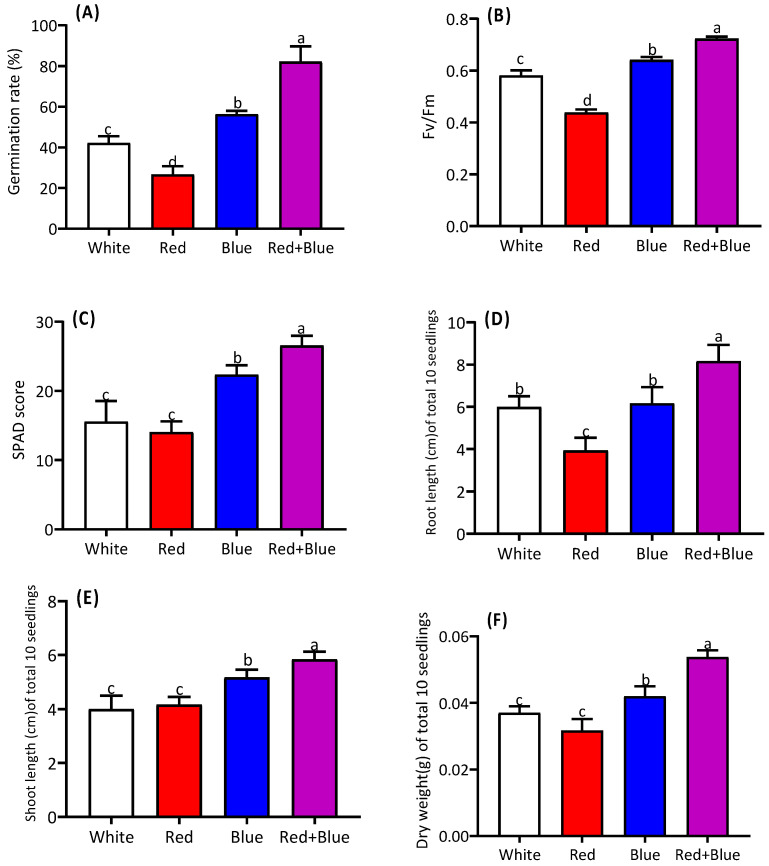
Regulation of morpho-physiological traits in alfalfa seedlings under different light qualities. Germination rate (**A**), Fv/Fm (**B**), SPAD score (**C**), root length (**D**), shoot length (**E**), and dry weight (**F**) of alfalfa seedlings grown under white, red, blue, and a combination of red and blue light (red+blue) are shown. Different letters on the bar columns indicate significant differences (*p* ≤ 0.05) among treatment means. At least three or more independent biological replications were considered during statistical analyses.

The light quality positively influenced the seed germination and morphological and physiological traits (Figure 1A–D) of the studied plants. Among the four types of light treatment, the red and blue (1:1) light resulted in the highest germination percentage (Figure 2A). The combination of red and blue light also showed the highest performance in the fluorescence of photosystem II (Fv/Fm) compared to the single treatments with white, red, or blue lights (Figure 2B). The leaf greenness, measured as a SPAD score, was also the highest under the combined red and blue light (Figure 2C). The root and shoot lengths, as well as the seedling dry weight, showed significant differences among the four treatments, with the combined light exposure yielding the longest root and shoot lengths (Figure 2D–E). Similarly, the combined light treatment significantly enhanced the seedling dry weight (Figure 2F).

### 2.2. Light Quality Enhanced Chlorophyll and Carotenoid Content in Leaves

**Figure 3:**
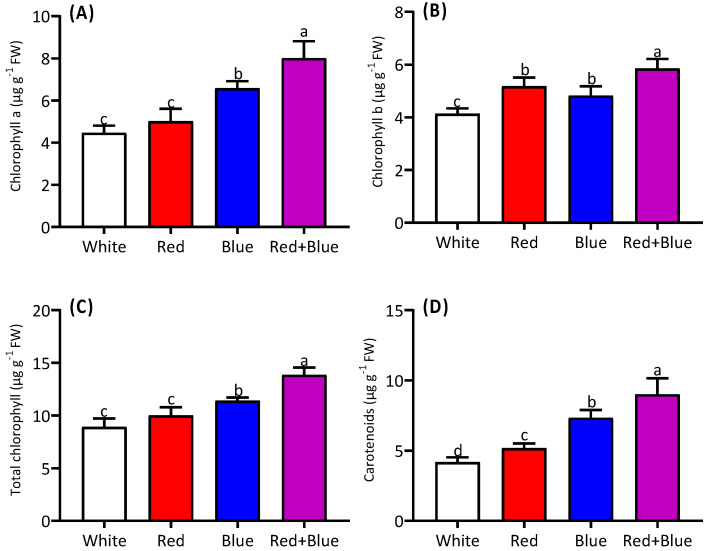
Improvement in photosynthetic pigments in alfalfa leaves under different light qualities. Chlorophyll a (**A**), chlorophyll b (**B**), total chlorophyll (**C**), and carotenoids content (**D**) in alfalfa seedlings grown under white, red, blue, and red combined with blue light (red+blue) are presented. Different letters on the bar columns indicate significant (*p* ≤ 0.05) differences among treatment means. At least three independent biological replications were considered during statistical analyses.

The light quality enhanced the chlorophyll and carotenoid contents in alfalfa seedlings (Figure 3). The chlorophyll content was higher under the combined red and blue light treatment (Figure 3A) than under white light with a single blue or red light. The chlorophyll *b* levels were also elevated under the combined red and blue light, with no significant difference being found between the separate red and blue light treatments (Figure 3B). Consequently, the total chlorophyll content was highest in the combined light treatment (Figure 3C). The carotenoid content varied significantly among the four treatments, with the combined red and blue light resulting in the highest carotenoid levels in the alfalfa seedlings (Figure 3D).

### 2.3. Light Quality Regulated ROS Signals

**Figure 4:**
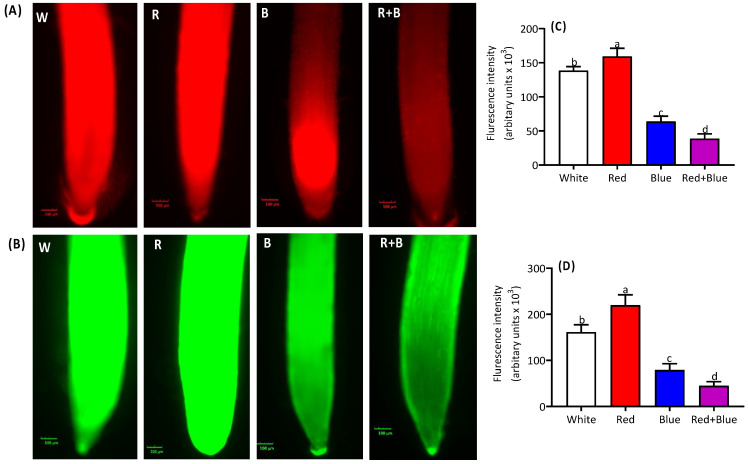
Regulation of ROS signals in alfalfa seedling roots under different light qualities. The O_2_^•−^ (**A**) and H_2_O_2_ signals (**B**) were observed on the fluorescence microscope. Graphical presentation of fluorescence intensity of O_2_^•−^ (**C**) and H_2_O_2_ (**D**) is provided. Alfalfa seedlings were grown and exposed under white, red, blue, and red combined with blue light (red+blue). Details of ROS (O_2_^•−^ H_2_O_2_)-specific probes and staining steps are presented in the Section 4. Different letters on the bar columns indicate significant (*p* ≤ 0.05) differences among the treatment means. At least three independent biological replications were considered during statistical analyses.

The superoxide (O_2_^•−^) and hydrogen peroxide (H_2_O_2_) signals in the studied alfalfa plants were regulated by different light exposures. The highest levels of O_2_^•−^ and H_2_O_2_ signals were observed under the red light (Figure 4A,B). We determined the fluorescence intensities of O_2_^•−^ and H_2_O_2_ in the studied plants, and the highest fluorescence efficiency of O_2_^•−^ was found in alfalfa root tips under red light, while the lowest intensity was found in the plants exposed to the combined red and blue lights (Figure 4C). Similarly, the H_2_O_2_ fluorescence patterns followed the same trend, showing significant differences across the light treatments (Figure 4D).

### 2.4. Differential Regulation of Antioxidant Enzyme Activity

**Figure 5:**
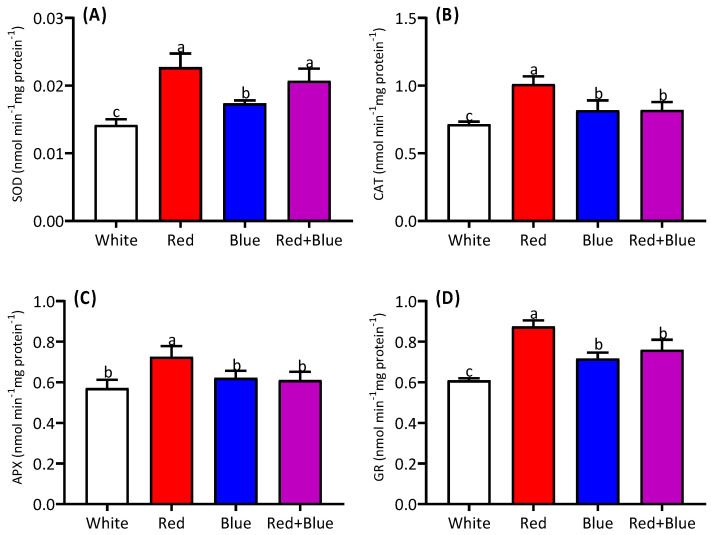
Alteration of antioxidant enzyme activity in alfalfa seedlings under different light qualities. Activities of SOD (**A**), CAT (**B**), APX (**C**), and GR (**D**) in alfalfa seedlings grown under white, red, blue, and red combined with blue light (red+blue). Different letters on the bar columns indicate significant (*p* ≤ 0.05) differences among the treatment means. At least three independent biological replications were considered during statistical analyses. Abbreviations: SOD, superoxide dismutase; CAT, catalase; APX, ascorbate peroxidase, GR, glutathione reductase.

The observed SOD, CAT, APX, and GR activities exhibited distinct regulatory patterns in response to various light exposures (Figure 5). The highest SOD activity was observed under the red light and the combined red and blue light treatment in the studied alfalfa (Figure 5A). Although no significant difference in SOD activity was observed between the red light and the combined (red+blue) treatments, the activity was slightly lower in the combined treatment. The highest level of CAT activity was observed under the red light compared to that under the white light, while no significant difference was detected between the blue light and the combined treatments (Figure 5B). No significant difference in the APX activity was found, except under red light exposure (Figure 5C). The GR activity was highest under the red light compared to the white light, with no significant difference being detected between the blue light and the combined (red+blue) treatments (Figure 5D).

### 2.5. Light Quality Regulated Candidate Gene Expression

**Figure 6:**
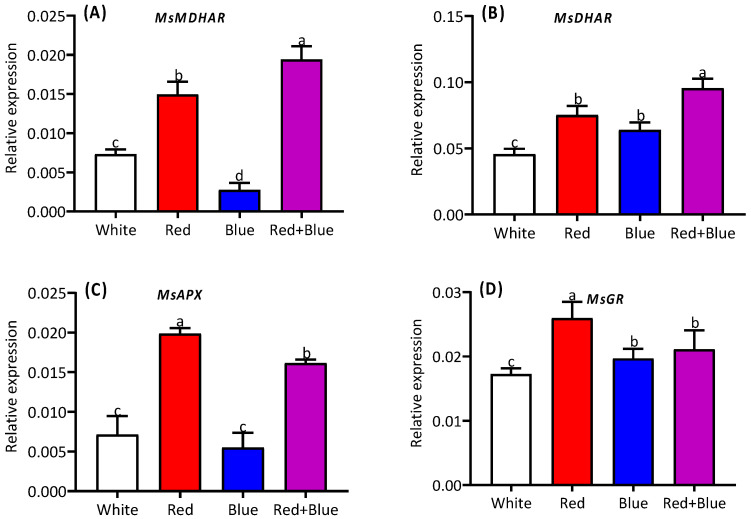
Alteration of candidate gene expression in alfalfa seedlings under different light qualities. Expression levels of *MsMDHAR* (**A**), *MsDHAR* (**B**), *MsAPX* (**C**), and *MsGR* (**D**) in alfalfa seedlings grown under white, red, blue, and red combined with blue light (red+blue). Different letters on the bar columns indicate significant (*p* ≤ 0.05) differences among the treatment means. At least three independent biological replications were considered during statistical analyses.

Different light qualities influenced the expressions of the candidate genes (Figure 6). The highest expression level of *MsMDHAR* among the four treatments was found in response to the combined (red+blue) light (Figure 6A). A similar expression pattern was found for *MsDHAR*, with no significant difference between the single red and blue light treatments, both of which showed higher expression levels compared to the white light treatment (Figure 6B). The expression of *MsAPX* was distinctly induced by the red and combined (red+blue) light (Figure 6C) treatments. The highest expression level of *MsGR* was observed under red light, while no significant difference was found between the blue and combined (red+blue) treatments for *MsGR* expression (Figure 6D).

### 2.6. Light Triggered the Nutrient Acquisition

**Figure 7:**
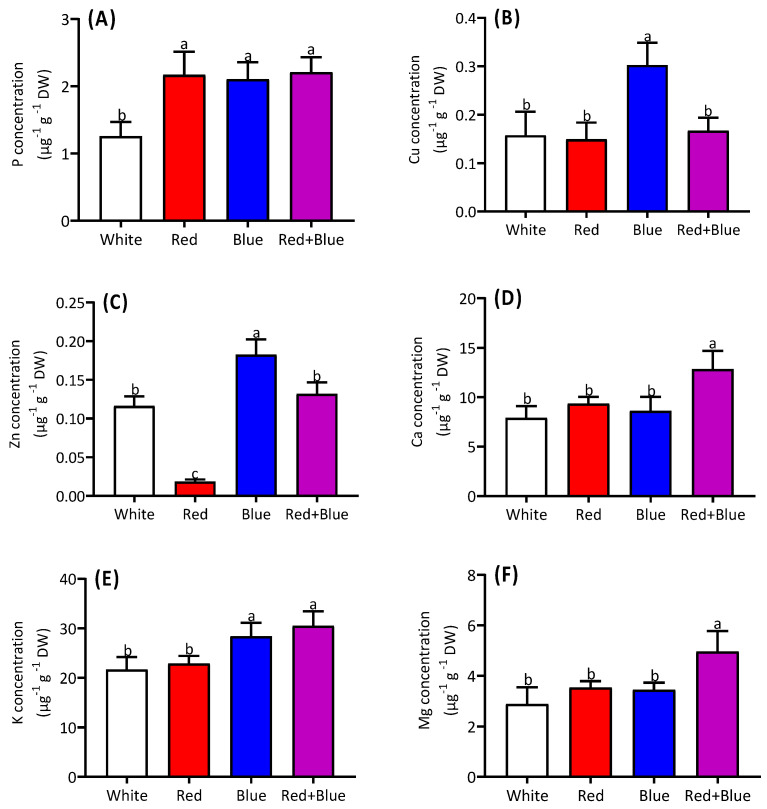
Regulation of mineral nutrients in alfalfa seedlings under different light qualities. Concentrations of P (**A**), Cu (**B**), Zn (**C**), Ca (**D**), K (**E**), and Mg (**F**) in alfalfa seedlings grown under white, red, blue, and red combined with blue light (red+blue). Different letters on the bar columns indicate significant (*p* ≤ 0.05) differences among the treatment means. At least three independent biological replications were considered during statistical analyses.

The red, blue, and combined (red+blue) light treatments exhibited a consistent pattern of phosphorus (P) acquisition in alfalfa seedlings (Figure 7A). The highest copper (Cu) acquisition was observed under the blue light compared to the other three treatments (Figure 7B). Similarly, the highest Zn acquisition was observed under the blue light, while no significant difference was observed between the white light and combined (red+blue) light treatments (Figure 7C). The calcium (Ca), potassium (K), and magnesium (Mg) concentrations were notably enhanced, particularly under the combined (red+blue) light treatments (Figure 7D–F).

## 3. Discussion

### 3.1. Light Quality Involved in Alfalfa Improvement

Most plant species absorb light within the 400–500 nm range for blue light and within the 600–700 nm range for red light, aligning with the peak absorption ranges of photoreceptors such as phototropins, phytochromes, and cryptochromes [19]. Red light is a critical factor that enhances seed germination and overall plant development [9]. In addition, red light stimulates plant growth regulators that promote increased cell division, elongation, stem length, and plant development [20]. In this study, we found that a combination of red and blue light (650 nm:450 nm; 1:1) significantly improves seed germination percentages (%), suggesting that combined light positively affects alfalfa seed germination. A previous study showed that blue light enhances stevia seed germination [21]. Similarly, our findings indicate that the combined light treatment used in this study is even more beneficial for alfalfa seed germination. Chlorophylls with carotenoids bind to particular apoproteins, forming light-harvesting and energy-transforming pigments in plants and other photosynthetic organisms [22]. Carotenoids play a key role in the photosynthesis process, and also assist as light-harvesting pigments and defend chlorophylls from photodestruction [22]. These findings suggest that the coordination of photosynthetic pigments is vital to the photosynthesis process. In our current study, we observed that combined red and blue light enhances germination, photosynthetic efficiency, and plant improvement of alfalfa plants. These findings have the potential to benefit low-germination plant species and support light-quality-based improvements in the growth and development of alfalfa and other forage legume species.

### 3.2. Light Responsive ROS Turnover

ROS generation during the photosynthetic electron transport process is a frequent event in plants. Additionally, the ROS signal is triggered in plants in response to various light qualities and spectra. This ROS generation and turnover varies with the light exposure time and light intensity (LI). High exposure for 3–6 h significantly increased the O_2_^•−^ levels in *Arabidopsis* and pea plants, while prolonged light exposure elicited similar ROS level patterns in wheat. Conversely, light exposure for 30 min triggered H_2_O_2_ production, a short-term ROS response known as a ROS wave, in *Arabidopsis* leave. H_2_O_2_ plays a vital role in signaling, while O_2_^•−^ and _1_O_2_ contribute to regulating the transcripts of candidate genes in *Arabidopsis* plants. These studies indicate that the light intensity regulates ROS turnover in plants. In this study, we observed that ROS signals are triggered by varying light intensities, with red light followed by white light generating the highest levels of ROS signals (O_2_^•−^ and H_2_O_2_). Red light generated the highest ROS levels, while the combination of blue and red light considerably reduced the ROS levels. These findings suggest that combined light (red+blue) is suitable for plants in a redox environment. These findings open new horizons for understanding the light-induced regulation of ROS waves in alfalfa and other forage legume species.

### 3.3. Light Quality Regulated Antioxidant Enzymes and Corresponding Genes

High light intensity-induced ROS generation is regulated by the activation of the antioxidant system. In this context, the activation of the antioxidant system in response to light quality has been reported in various plant species, including the model plant *Arabidopsis* [23] and wheat [24]. The induction of antioxidant genes (POD, APX, SOD, and CAT) results from transcriptional regulation in response to varying light qualities [6]. Therefore, it is evident that ROS signal induction, oxidative stress, and antioxidant enzyme activity are interconnected. The induction of ROS (H_2_O_2_) is not necessarily harmful. It has been reported that red light induces an H_2_O_2_ signal, providing resistance to powdery mildew in melon seedlings [25]. In response to ROS generation triggered by light exposure, plants have evolved an antioxidant system to homeostat ROS levels. In our current study, the elevation of SOD, CAT, APX, and GR under red light indicates that red light is involved in generating ROS signals in alfalfa seedlings. These findings regarding antioxidant enzymes align with red light-induced ROS signals observed through fluorescence microscopy. Interestingly, the ROS intensity was lower under combined light (red+blue), suggesting that the combined light treatment used in this study can redox the environment in alfalfa roots. SOD serves as the first line of defense, effectively combating oxidative stress [26,27]. Catalase is a key antioxidant enzyme that mitigates oxidative stress [28]. In this study, combined red and blue light induced CAT activity, suggesting that CAT was more responsive to red light, which also decreases cellular oxidative stress levels. APX plays a key role in redox homeostasis, where GR contributes to H_2_O_2_ elimination via the AsA–GSH cycle, by metabolizing H_2_O_2_ [29,30]. As a consequence, the corresponding gene expression was induced under red light. The elevated expression levels of the genes (*MsAPX*, *MsGR*) indicate their involvement in the elimination of light-induced ROS in alfalfa plants. The AsA–GSH cycle, involving other genes such as *MsMDHAR*, and *MsDHAR*, was highly expressed under red and combined light (red+blue), suggesting that these genes play a more active role in regulating the AsA accumulation and homeostasis of ROS in alfalfa plants. Our findings are consistent with previous research linking DHAR to light-induced ascorbate accumulation [31].

### 3.4. Light Quality Regulated Nutrient Acquisition in Alfalfa Plants

The quality of light induces nutrient regulation in various plant species. Numerous studies have revealed that red light and blue light alter the micro- and macro-nutrient acquisition in plants [32]. Blue light and green light improve plant growth and regulate Fe, Zn, Cu, Ca, and Se, although they do not affect Mn and P acquisition in plants [15,33]. Blue light induces Fe, Zn, Cu, and Mg acquisition in lettuce [16]. In addition to single-light treatment, combined light (blue+red) influences nutrient acquisition in plants. For instance, the red and blue light (R:B; 1:1) used in this study enhances the P, S, B, Ca, Mg, Cu, Fe, Zn, Mn, and Mo acquisition in *Brassica oleacea* [17]. In our study, we observed that the P, Cu, Zn, Ca, K, and Mg levels in alfalfa increased significantly, particularly in response to blue light or a combination of red and blue (1:1) light. Furthermore, the combined red and blue light enhanced the concentrations of Ca, K, and Mg to their highest levels. The variations in the mineral content under various light conditions suggest that the light ratio is one of the key factors that influences the acquisition of minerals in plants.

## 4. Materials and Methods

### 4.1. Plant Cultivation and Quality Light Exposure

Healthy, viable seeds of alfalfa (*Medicago sativa* L.) were rinsed three times with sterilized water and then placed in a germination tray in the dark for 72 h. Alfalfa seedlings were cultivated in a one-fourth-strength Hoagland nutrient solution and exposed to various light qualities: white (400 nm), red (660 nm), blue (450 nm), and blue combined with red (650 nm:450 nm; 1:1). The seedlings were maintained in a controlled growth environment for 2 weeks. The growth chamber was maintained at a temperature of 25 °C with a relative humidity of 50–55%. After two weeks of continuous exposure to different light qualities, the seedlings were carefully separated and harvested. The alfalfa seedling roots were rinsed with distilled water and placed on tissue paper to remove excess moisture. The seedlings were then packed in small polybags, flash-frozen with liquid nitrogen, and stored at −80 °C for further analysis.

### 4.2. Analyses of Morphological and Physiological Traits

The germination percentage (%) of alfalfa seeds was calculated by dividing the number of germinated seeds by the total number of seeds planted and multiplying the result by 100. The maximum fluorescence of photosystem II (Fv/Fm) in alfalfa leaves was measured using a fluorometer (PAM-2100, Effeltrich, Germany). The chlorophyll content of alfalfa leaves was determined based on leaf greenness (SPAD value) using a chlorophyll meter (SPAD-503, Manitoba, Japan). The root and shoot lengths of seedlings were measured with a centimeter (cm) scale and their dry weight (g) was determined using a digital balance.

### 4.3. Determination of Chlorophyll and Carotenoids

Alfalfa chlorophyll content was determined following the protocol described by Ritchie et al. [34]. Briefly, 400 mg of ground alfalfa sample was homogenized with a dimethyl sulfoxide (DMSO: 99.5%) solution (Sigma-Aldrich, St. Louis, MO, USA). The homogenate was then heated in a dry oven at 65 °C for 3 h. After incubation, the mixture was centrifuged at 10,000 rpm for 10 min. The same isolated extract was used for carotenoid determination. The supernatant was transferred to a new Eppendorf tube, and its absorbance was measured at 452, 644, and 663 nm. Chlorophyll a and b and carotenoid content were calculated using the following formulas:

Chlorophyll a ((µg)⁄g) = 10.3 × A663 − 0.918 × A644;

Chlorophyll b ((µg)⁄g) = 19.7 × A644 − 3.878 × A644;

Total chlorophyll = Chlorophyll a + Chlorophyll b;

Carotenoids ((µg)⁄g) = 4.2 × A452 × (0.0264 × Chlorophyll a + 0.426 × Chlorophyll b.

### 4.4. Detection of ROS (H_2_O_2_ and O_2_^•−^) Signals

Approximately 1–2 mm sections of alfalfa root tips were cut and rinsed with deionized water. The ROS-specific fluorescence probe was prepared using 10 mM Tris–HCl buffer (pH 7.4). The root tips were incubated at 37 °C for 30 min in the dark with 10 μM dihydroethidium (DHE) and then gently rinsed with the same buffer. Reactive oxygen species (O_2_^•−^) signals were detected at excitation and emission wavelengths of 488 nm and 520 nm, respectively. The same initial procedure was followed for H_2_O_2_ detection, but in this case, a specific probe, 2′,7′-dichlorofluorescin diacetate (DCF-DA, Sigma-Aldrich), was used. The samples were incubated with 25 μM DCF-DA for 30 min. Afterward, the root samples were washed with the same buffer, and the excitation and emission were recorded at 480 nm and 530 nm, respectively [35]. The probe-treated root tips were washed twice with DEPC-treated water and mounted on a glass slide, and H_2_O_2_ signals were detected using a fluorescence system (CLS-01-00076, Logos Biosystem, Anyang, Kyonggi-do, Republic of Korea).

### 4.5. Measurement of Antioxidant Enzymes Activity

Antioxidant enzymes including superoxide dismutase (SOD), catalase (CAT), ascorbate peroxidase (APX), and glutathione reductase (GR) were extracted following a previously described protocol [36]. Briefly, 200 mg of alfalfa plant tissue was homogenized with potassium phosphate (KP) buffer (100 mM, pH 7.0). The homogenate was then centrifuged at 13,000 rpm for 10 min and the resulting extract was used to measure enzyme activity. SOD activity was measured based on experimental steps and chemical concentrations that were previously recommended [37]. CAT activity was determined using a step-point-by-point method described earlier [38]. For this, 100 µL of the plant extract was mixed with 0.1 mM EDTA, 50 mM KP buffer, 0.1 mM hydrogen peroxide, and 0.5 mM ascorbate. The absorbance and subsequent calculations were performed as outlined in a prior protocol [35]. To measure GR activity, 100 µL was combined with 0.2 M KP buffer, 1mM EDTA, 20 mM GSSG, and NADPH. Readings and calculations of the extinction coefficient (6.12 mM^−1^ cm^−1^) were conducted following the method described by Halliwell and Foyer [39].

### 4.6. Analysis of Candidate Gene Expressions

To analyze gene expression, total RNA was extracted from two-week-old alfalfa seedlings using the RNeasy^®^ plant mini kit (QIAGEN, Hilden, Germany). Briefly, 300 mg of alfalfa tissue was mixed with a buffer containing DDT (2M) and 1% (*v*/*v*) 2-mercaptoethanol. The mixture was vortexed and then centrifuged at 13,000 rpm for 2 min. RNA was collected through multiple washing and filtering steps. The total RNA concentration was determined using a micro-drop system (UVISDrop-99, Taipei, Taiwan). Candidate gene-specific primers were designed for these experiments (Supplementary Table S1). Gene expression was analyzed using a real-time PCR system (CFX96, Bio-Rad, Hercules, CA, USA). The qPCR reactions (20 µL each) were prepared using a combination of SYBR green, RNA sample, buffer, and enzymes following the manufacturer’s instructions (SYBR green, Bioneer, Daejeon, Republic of Korea). *MsActin* was considered an as the internal control, and candidate gene expression levels were calculated using the 2^−ΔΔCt^ method [40]. Each calculation was based on at least three independent replicates.

### 4.7. Measurement of Elemental Concentrations

Alfalfa seedlings were dried in a hot oven at 80 °C for 72 h. The dried samples were weighed and treated with HNO_3_/HClO_4_ (3:1, *v*/*v*) for 8 h before digestion. The samples were then heated and digested using the same solution, HNO_3_/HClO_4_ (3:1, *v*/*v*), following previously established protocols [41]. Elemental concentrations were measured using an inductively coupled plasma mass spectrometer (ICP-MS, Agilent 7700, Santa Clara, CA, USA) with metal-specific standard solutions based on a reference curve.

### 4.8. Statistical Analyses

All physiological and molecular data were analyzed using analysis of variance (ANOVA). Significant differences (*p* < 0.05) among the group mean were determined using a *t*-test. GraphPad prism (Version 9.0) was used to create graphical presentations. At least three independent biological replications were considered for data analyses.

## 5. Conclusions

**Figure 8:**
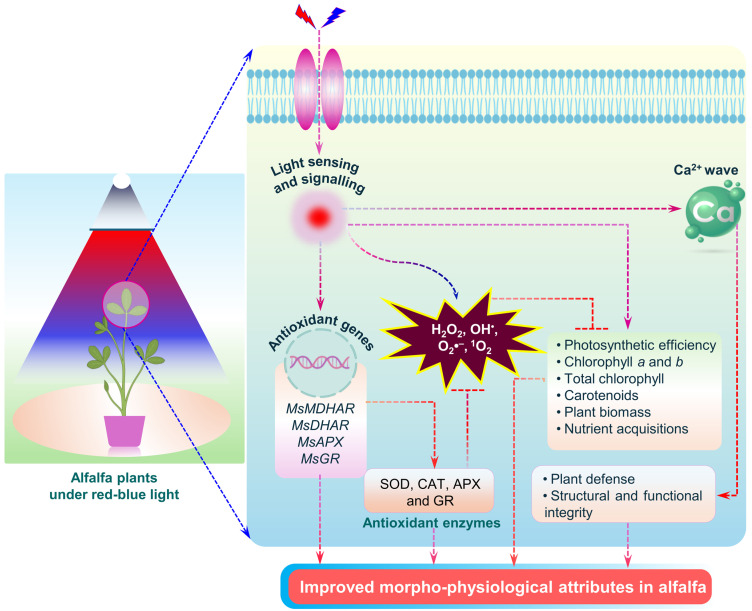
Mechanical insights of light-induced alfalfa improvement under different light qualities. The blue or combined red and blue light induces a series of physiological and molecular alterations in alfalfa. During these processes, light sensing and signaling trigger signaling molecules (ROS, Ca^2+^). Light-induced ROS signals inhibit the photosynthetic efficiency, synthesis of photosynthetic pigments (chlorophyll a, chlorophyll b, carotenoids), plant biomass production, and nutrient acquisition. In contrast, Ca^2+^ contributes to plant defense as well as structural and functional integrity. At the molecular level, light induces the expression of candidate genes (*MsMDHAR*, *MsDHAR*, *MsAPX*, and *MsGR*) and key enzymes (SOD, CAT, APX, and GR), which leads to inhibiting excess ROS (O_2_^•−^, H_2_O_2_) generation. These combined physiological and molecular alterations enhance morphophysiological traits in alfalfa plants. Abbreviations: ROS, reactive oxygen species; MDHAR, monodehydroascorbate reductase; DHAR, dehydroascorbate reductase; APX, ascorbate peroxidase; GR, glutathione reductase; SOD, superoxide dismutase; CAT, catalase; and Ca, calcium.

This study explores mechanistic insights into the light quality-induced regulation of seed germination, photosynthetic efficiency, ROS signaling, antioxidant enzyme activity, candidate gene expression, and mineral nutrient acquisition in alfalfa (Figure 8). The combination of red and blue light (1:1) improved alfalfa seed germination and its morphological and physiological attributes. These findings suggest that the effect of the combined light used in this study is suitable for improving alfalfa plants. The red light-induced ROS signaling observed in this study was mitigated by antioxidant enzymes and their corresponding genes, suggesting the increased activity of these enzymes and genes during light-induced ROS homeostasis. This study further suggests that red or combined red and blue light is effective in enhancing mineral acquisitions in alfalfa seedlings. These findings collectively open new horizons for the quality light-induced improvement of alfalfa and other forage legumes.

## Data Availability

Please contact the corresponding author for any additional information.

## Supplementary Materials

The following supporting information can be downloaded at: https://www.mdpi.com/article/10.3390/ijms26010360/s1.
