# Supplementary material for: Light Quality Plays a Crucial Role in Regulating Germination, Photosynthetic Efficiency, Plant Development, Reactive Oxygen Species Production, Antioxidant Enzyme Activity, and Nutrient Acquisition in Alfalfa
Source: Int J Mol Sci. 2025 Jan 3;26(1):360. doi: 10.3390/ijms26010360 (PMC11720064; doi:10.3390/ijms26010360)
Supplement: Supplementary file 1 [file ijms-26-00360-s001.zip › ijms-3287976-supplementary.pdf]

Supplementary Table S1: *Medicago* primer sequences used for this study.

**Gene name**	**Forward**	**Reverse**	**Accession**
ACTIN	TTCTCACCACACTTCTCGCC	CCAGCCTTCACCATTCCAGT	JQ028730.1
MDHAR	TGGTCTACCCAGAACCTTGG	CCAGTACCCTTCCGTCCTTT	EX522162.1
DHAR	GTGTTGCTGACACTGGAGGA	CCAGCTGTAGCCTTTTCAGG	DQ006811.1
APX	GAAATGCGCTCCTCTTATGC	TGTTAGCACCATGAGCAAGC	EX522382.1
GR	AGGACGGTGAACCTGATTTG	TCTAGCAGCACGAACACCAC	AM407889.2
